# From aging to long COVID: exploring the convergence of immunosenescence, inflammaging, and autoimmunity

**DOI:** 10.3389/fimmu.2023.1298004

**Published:** 2023-10-24

**Authors:** Ludmila Müller, Svetlana Di Benedetto

**Affiliations:** Max Planck Institute for Human Development, Center for Lifespan Psychology, Berlin, Germany

**Keywords:** aging, immunosenescence, autoimmunity, inflammaging, epigenetics, SARS-CoV-2, long COVID

## Abstract

The process of aging is accompanied by a dynamic restructuring of the immune response, a phenomenon known as immunosenescence. This mini-review navigates through the complex landscape of age-associated immune changes, chronic inflammation, age-related autoimmune tendencies, and their potential links with immunopathology of Long COVID. Immunosenescence serves as an introductory departure point, elucidating alterations in immune cell profiles and their functional dynamics, changes in T-cell receptor signaling, cytokine network dysregulation, and compromised regulatory T-cell function. Subsequent scrutiny of chronic inflammation, or “inflammaging,” highlights its roles in age-related autoimmune susceptibilities and its potential as a mediator of the immune perturbations observed in Long COVID patients. The introduction of epigenetic facets further amplifies the potential interconnections. In this compact review, we consider the dynamic interactions between immunosenescence, inflammation, and autoimmunity. We aim to explore the multifaceted relationships that link these processes and shed light on the underlying mechanisms that drive their interconnectedness. With a focus on understanding the immunological changes in the context of aging, we seek to provide insights into how immunosenescence and inflammation contribute to the emergence and progression of autoimmune disorders in the elderly and may serve as potential mediator for Long COVID disturbances.

## 1 Introduction

The inevitable passage of time brings with it a remarkable series of physiological transformations collectively known as aging. Within these changes, our immune system undergoes a complex remodeling, a phenomenon summarized by the term “immunosenescence” (1–3). Central to this phenomenon is a progressive alteration in immune system dynamics as a consequence of aging (1, 4–6). Recent empirical evidence has illuminated the physiological underpinnings of immunosenescence, which is now understood as an adaptive alteration of the immune system within the context of the aged microenvironment, rather than a mere collapse of the system itself (6, 7). Manifesting as shifts in immune cell composition and functional characteristics, immunosenescence frames the contextual background within which the consequent interactions occur (8).

Simultaneously, the emerging orchestration of chronic inflammation, or “inflammaging,” gains prominence (9). A complex interplay involving inflammasomes, cytokines, and senescent cells contributes to a persisting pro-inflammatory milieu that underlines age-associated immune shifts. These age-related alterations in immune dynamics and inflammatory responses have far-reaching consequences, one of which is the altered predisposition to autoimmune diseases among older individuals (6, 10). Epigenetic changes, integral to gene expression regulation, have emerged as key modulators of immune responses and aging (6, 11, 12). The dynamic interplay between DNA methylation, histone modifications, and non-coding RNA regulation not only influences immune cell function but also plays a role in shaping susceptibility to infections and the development of autoimmune conditions (6, 11, 13, 14).

In the sections that follow, we will define the key components of immunosenescence, delve into the nuances of inflammaging and its consequences, explore the altered landscape of autoimmunity in the elderly, and reveal the potential molecular and cellular mechanisms that bridge these phenomena. The importance of understanding these complex interactions lies not only in advancing our understanding of the fundamental processes governing immune system function. By unraveling the relationship of immunosenescence, inflammation, and autoimmunity, we pave the way for developing strategies that target age-associated immune and post COVID dysregulation and hold promise for enhancing the quality of life for the elderly.

## 2 Definition and key features of immunosenescence

Immunosenescence, an intricate term coined to describe the aging of the immune system, summarizes a series of multifaceted changes that collectively influence immune function over time (6, 7). This phenomenon reflects a gradual alteration in the immune response dynamics, resulting in a less efficient and coordinated defense against various challenges. Key features of immunosenescence encompass shifts in immune cell populations, functional adaptations, and altered signaling pathways, all of which contribute to the overall decline in immune competence with age (15, 16).

**Figure 1:**
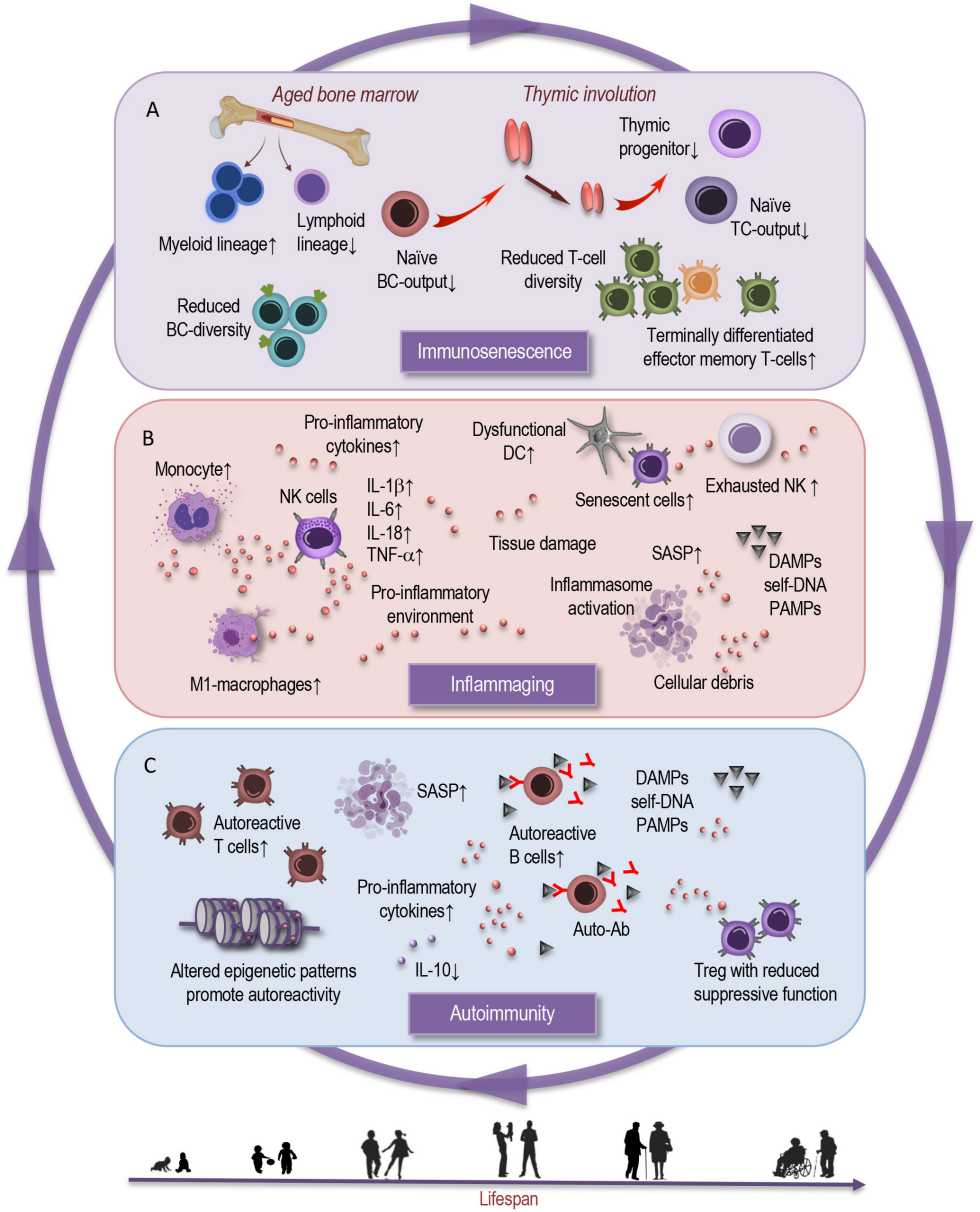
Interplay between immunosenescence, inflammaging, and autoimmunity. This simplified scheme illustrates the reciprocal interactions that characterize interdependent processes of immunosenescence, inflammation, and autoimmunity. **(A)** Immunosenescence is marked by distinct shifts in immune cell populations and decline in immune responsiveness, impaired T-cell function, compromised B- and T-cell diversity. Age-dependent physiological and functional alterations in the bone marrow and thymic involution contribute to shifts in the myeloid and lymphoid lineages, and in proportions of naive and memory T cells. **(B)** Parallelly, the orchestration of chronic inflammation, emerges as a defining backdrop against which these immunological changes unfold. This chronic inflammatory milieu – inflammaging - fuels and is fueled by the aging immune milieu and is intertwined by age-dependent alterations in innate immune cell subsets, in inflammasomes, cytokines, and cellular senescence. These bidirectional age-related processes may serve as both a catalyst and an offender of **(C)** autoimmunity. Age-associated changes in Treg frequency and function can result in the ineffective suppression of autoreactive T cells, causing autoimmune responses. Altered epigenetic patterns promote autoreactivity. In the broader context, epigenetic modifications choreograph the interplay between immune cell subsets, inflammatory reactions, and may contribute to potential dysregulations. The cumulative effect of these processes manifests in the emergence and progression of age-related autoimmune disorders.BC, B cell; TC, T cell; DC, dendritic cell; NK, natural killer cell; SASP, senescence associated phenotype; DAMPs, damage associated molecular patterns; PAMPs, pathogen associated molecular patterns; IL, interleukin; Auto-Ab, autoreactive antibody; Treg, regulatory T cell.

A hallmark of immunosenescence is the remodeling of immune cell populations, which play pivotal roles in orchestrating immune responses (6, 8, 16). The cells of adaptive immunity undergo remodeling during the aging process characterized by a loss of receptor diversity and compromised immunological memory formation. Central to immunosenescence of adaptive immunity (**Figure 1A**) are thymic involution and insufficient hematopoietic stem cell function (17–19). Generally, older individuals exhibit decreased responsiveness to new antigens due to a decline in the generation of naive T cells from the thymus (19–22). There is a decline in the diversity of the T-cell receptor repertoire, which compromises the ability to recognize a broad array of antigens (6, 16). Moreover, the balance between naive and memory T cells tilts, leading to a reduced capacity to deal with new antigens while potentially favoring autoimmune reactions (14, 23).

Nonetheless, homeostatic proliferation partly sustains the diversity of the T-cell receptor repertoire (24). Furthermore, peripheral T cells in the elderly typically exhibit reduced absolute numbers, an altered CD4:CD8 ratio, and an expansion of terminally differentiated effector memory T cells (22, 25). These changes are correlated with impaired proliferative capacity, reduced telomerase activity, and compromised intracellular signaling (24, 26, 27). Additionally, a substantial proportion of regulatory T lymphocytes in adults represent a terminally differentiated, functionally impaired population prone to apoptosis, possessing limited self-renewal potential (16, 27, 28). This observation may explain, at least in part, the emergence of autoimmune conditions associated with aging.

B cells, responsible for antibody production, also exhibit age-related alterations (**Figure 1A**). The production of new B cells in the bone marrow diminishes, and the repertoire of antibody specificity becomes narrower (6, 29–33). These shifts contribute to reduced immune surveillance against novel pathogens and a diminished vaccine responses in the elderly. It is notable that the development of antibodies, including those induced by vaccines, is profoundly affected by aging, as supported by the findings of Frasca et al. (31). The study indicates that although the frequencies of influenza vaccine-specific memory B cells and plasmablasts were comparable between young and elderly individuals, the elderly exhibited a reduced fold-increase in serum titers after vaccination (31). This suggests that not only is there a decrease in the production of new B cells at the bone marrow level but also that the B cells produced in aging individuals appear to have diminished responsiveness to vaccinations, resulting in a compromised antibody response. Consequently, the elderly may exhibit reduced efficacy in generating protective antibodies even after immunization, highlighting a critical aspect of immunosenescence that may impact vaccine effectiveness and overall immune resilience.

Natural killer (NK) cells, critical for innate immune defense, experience functional shifts. A subset of NK cells with heightened cytotoxicity declines, while a subset with regulatory functions increases, possibly negatively affecting the immune response against infections and malignancies (**Figure 1B**) (4, 6, 34–36). Myeloid cells, encompassing various immune cell types, like monocytes, macrophages, and dendritic cells, undergo changes that influence their antigen-presenting capabilities and enhance the pro-inflammatory tendencies (6, 35, 37, 38). This altered landscape (35, 39) can disrupt the delicate balance between immune activation and regulation.

In summary, immunosenescence is marked by distinct shifts in immune cell populations and a decline in immune responsiveness. Impaired T-cell function, compromised B- and T-cell diversity, and altered NK-cell cytotoxicity collectively contribute to a weakened defense against pathogens. Furthermore, the imbalance between innate and adaptive immunity may disrupt the precise regulation of effector immune responses, potentially resulting in a pronounced pro-inflammatory state.

## 3 Chronic low-grade inflammation in aging

Like the faint hum of background noise, chronic low-grade inflammation - termed “inflammaging” - emerges as a hallmark of the aging process (10, 40). This phenomenon refers to a persistent, low-grade pro-inflammatory state that permeates various tissues and organs throughout the lifespan (41, 42). Unlike the acute inflammation that arises in response to infections or injuries, inflammaging lacks a clear stimulus and is driven by a complex interplay of molecular and cellular events. This chronic inflammatory milieu is implicated in the pathogenesis of numerous age-related disorders, including cardiovascular diseases, neurodegenerative conditions, metabolic and autoimmune disorders (10, 17, 40, 43).

The orchestration of inflammaging involves a convergence of factors operating at both the molecular and cellular levels (**Figure 1, B**). Inflammasomes, intracellular multiprotein complexes that facilitate the activation of pro-inflammatory cytokines, are pivotal players in this process (10, 40, 44). They serve as intracellular sensors for damage-associated molecular patterns (DAMPs) and pathogen-associated molecular patterns (PAMPs), triggering the maturation and secretion of pro-inflammatory cytokines like interleukin-1β (IL-1β) and IL-18 (4, 45). The chronic activation, driven by cellular stress and accumulation of cellular debris, contributes to sustained inflammation in aging tissues. Cytokines such as IL-6 and tumor necrosis factor-alpha (TNF-α) are key mediators in propagating the inflammatory response. Elevated levels of these cytokines are associated with aging and are believed to be central in orchestrating inflammaging (6, 10, 44).

Another significant contributor to inflammaging is cellular senescence – a state of irreversible cell cycle arrest accompanied by a pro-inflammatory secretory phenotype known as the senescence-associated secretory phenotype (SASP). Senescent cells accumulate with age and secrete various pro-inflammatory cytokines, chemokines, and growth factors that perpetuate the inflammatory milieu (**Figure 1, B**). These senescent cells not only contribute to local tissue inflammation but also have systemic consequences, fostering a microenvironment beneficial to age-related pathologies and autoimmune conditions (6, 46–48).

Inflammaging, with its widespread pro-inflammatory influence, is associated with a spectrum of age-related diseases (46, 49). It may contribute to oxidative stress, immune dysregulation, and endothelial dysfunction – key drivers of cardiovascular diseases (10, 50). Moreover, the pro-inflammatory environment generated by inflammaging can adversely affect neural health, contributing to neurodegenerative conditions like Alzheimer’s and Parkinson’s disease (6, 51). Inflammaging also promotes metabolic dysregulation by interfering with insulin signaling pathways and promoting adipose tissue inflammation (17, 52). This inflammatory backdrop amplifies the risk of metabolic disorders such as type 2 diabetes and obesity (53). Thus, inflammaging serves as an essential bridge between chronic low-grade inflammation and the onset of age-related diseases. Inflammatory cytokines can promote immune cell activation and tissue damage, exacerbating autoimmune diseases (15, 40, 42, 52, 54, 55).

Taken together, the interplay between inflammasomes, cytokines, cellular senescence, and the resulting inflammatory milieu underscores the significance of understanding these processes for deciphering the intricate network of aging-associated and autoimmune disorders.

## 4 Shifting immune tolerance and self-recognition with age

Immunosenescence and inflammaging can influence the onset, course, and severity of autoimmune diseases (56, 57). Autoimmunity arises from a breakdown in immune tolerance, leading to immune attacks against self-antigens (46). These disorders encompass a wide range of conditions, including rheumatoid arthritis, systemic lupus erythematosus, and multiple sclerosis. The compromised regulatory mechanisms and altered immune responses associated with aging, may contribute to the development and progression of autoimmune conditions. Immunosenescence can lead to dysregulation of self-tolerance by disrupting the delicate balance between self-tolerance and immune activation (47, 58, 59). Age-related thymic involution can impact central tolerance by reducing the elimination of self-reactive T cells in the early stages of development (**Figure 1A**). This can lead to increased numbers of autoreactive T cells in the periphery, raising the risk of autoimmune responses (6, 17, 59) (**Figure 1C**).

On the molecular level, aging imparts perturbations in T-cell receptor (TCR) signaling that contribute to autoimmunity (60). The diminished phosphorylation of key signaling molecules, such as ζ-chain-associated protein kinase 70 (ZAP-70), in aged T cells attenuates the strength and accuracy of TCR signaling. This phenomenon, coupled with a decline in TCR repertoire diversity, hampers the immune system’s ability to distinguish self from non-self (6, 60, 61). Consequently, autoreactive T cells can evade regulatory mechanisms, proliferate (**Figure 1C**), and contribute to autoimmune pathogenesis.

Also, regulatory T cells (Tregs), crucial for preventing excessive immune responses against self-antigens, exhibit reduced suppressive function in the aging immune system (**Figure 1C**). The reduction in Treg suppressive potency (28, 59, 62), coupled with age-related changes in Treg-associated transcription factors like Foxp3, diminishes their ability to restrain autoreactive T cells (63). This impairment of immunomodulatory control facilitates the expansion of self-reactive immune effectors and the progression of autoimmune responses.

B cells play a significant role in autoimmunity by producing autoantibodies and impacting B-cell tolerance (**Figure 1C**). Immunosenescence can disturb central and peripheral B-cell tolerance mechanisms. Defects in negative selection of immature B cells and alterations in B-cell receptor signaling contribute to the production of self-reactive antibodies, fostering autoimmune responses (6, 32, 58, 64).

Furthermore, immunosenescence can also impact immune checkpoint molecules, which are crucial for maintaining self-tolerance (6, 65, 66). Programmed cell death protein 1 (PD-1) and cytotoxic T-lymphocyte-associated protein 4 (CTLA-4) are key regulators of immune responses. With age, these checkpoints may be dysregulated, leading to increased activation of autoreactive T cells and contributing to autoimmune pathology.

Dysregulated cytokine production may also fuel autoimmunity by propagating chronic inflammation and facilitating the activation of self-reactive immune cells (**Figures 1B, C**). The increased production of pro-inflammatory cytokines, including IL-6 and IL-1β, amplifies the inflammatory milieu and exacerbates autoimmune responses (6, 67). Simultaneously, the downregulation of anti-inflammatory cytokines, such as IL-10, diminishes the immune system’s capacity to suppress autoimmune reactions. This imbalance tilts the equilibrium towards immune dysregulation and the propagation of autoimmunity (8, 49).

Thus, the etiology of autoimmunity is multifactorial, encompassing along with genetic susceptibility, also environmental triggers, and a complex interplay of immunoregulatory mechanisms. Dysfunctional clearance of senescent cells, SASP, and the resultant chronic inflammation interplay with age-related changes in immune homeostasis, maintaining a milieu conducive to autoimmune reactions.

## 5 Age-related epigenetic alterations contributing to autoimmunity

Epigenetic modifications play a pivotal role in orchestrating gene expression without altering the underlying DNA sequence. These modifications, including DNA methylation and histone modifications, act like molecular switches, determining whether a gene is turned on or off. Such dynamic mechanism enables cells to adapt to diverse environments and physiological states including aging itself (6, 11–13).

Aging is accompanied by a number of epigenetic changes that influence cellular function and identity. DNA methylation, the addition of methyl groups to DNA, tends to increase in certain genomic regions over time, resulting in altered gene expression patterns. Likewise, histone modifications, which influence how tightly DNA is wound around histone proteins, shift with age, impacting gene accessibility. Furthermore, non-coding RNAs, often overlooked in the past, are now recognized as key players in gene regulation, with their dysregulation linked to aging-associated changes (6, 11).

Epigenetic changes exert far-reaching effects on the immune landscape, having implications for immune responses, autoimmunity, and infection susceptibility (13). In the context of immune responses, they guide the differentiation of immune cells, influencing their functions and responsiveness (6, 68). Epigenetic modifications can tilt the balance toward immune activation or suppression, contributing to the immune dysregulation observed in aging and autoimmune conditions (**Figure 1C**). Furthermore, they can prime immune cells for specific responses, potentially rendering individuals more or less susceptible to infections and more or less predisposed to inflammatory conditions. Moreover, epigenetic shifts can fuel the flames of autoimmunity (6, 69). Altered epigenetic patterns can promote the production of self-reactive immune cells and foster immune system confusion between self and non-self. This phenomenon highlights the contribution of epigenetic changes to the development and progression of autoimmune diseases.

Taken together, epigenetic changes underscore the complexity of immune aging and the intertwined influences of aging and immune responses. Understanding how these epigenetic modifications direct immune cell behavior, impact autoimmune susceptibility, and influence infection outcomes (like COVID-19) is pivotal. It holds the promise of elucidating new therapeutic avenues to counteract age-related immune decline and promote immune balance, potentially alleviating the burden of autoimmune conditions and infections in aging individuals.

## 6 Autoimmunity as potential contributor to long COVID?

Long COVID, also known as post-acute sequelae of SARS-CoV-2 infection, is a complex and poorly understood condition that affects individuals who have recovered from the acute phase of COVID-19 but continue to experience a range of symptoms for weeks or even months afterward (70, 71). The role of autoimmunity in Long COVID is a topic of ongoing research, and our understanding of it is evolving (6, 71, 72). While the exact mechanisms behind Long COVID are not fully understood, there is growing evidence to suggest that autoimmunity may play a role in some cases. It is hypothesized that the immune system, having been initially activated to fight the SARS-CoV-2 virus during the acute infection, may become dysregulated and turn against the body’s own cells and tissues (15, 73, 74). This dysregulation can lead to chronic inflammation and a wide range of symptoms. Here are some key aspects supporting the idea of an autoimmune component in Long COVID:

- **Autoantibodies**: Some Long COVID patients have been found to produce autoantibodies, which can target the body’s own tissues or proteins. These autoantibodies can contribute to inflammation and tissue damage, resembling the autoimmune response seen in conditions like lupus or rheumatoid arthritis.
- **Persistent inflammation**: Chronic inflammation is a hallmark of many autoimmune diseases. In Long COVID, there is often evidence of ongoing inflammation, which can affect various organs and systems in the body. This chronic inflammation may be driven by an immune response that remains active long after the initial viral infection has cleared.
- **Autoimmune-like symptoms**: Many individuals with Long COVID report symptoms that are reminiscent of autoimmune diseases. These symptoms may include chronic fatigue, joint and muscle pain, brain fog, skin rashes, and other systemic issues. The presence of these symptoms, which can persist for weeks or months, suggests a potential dysregulation of the immune system. The similarity in symptoms raises the possibility of shared immunological mechanisms.
- **Immunosuppressive treatments**: In some cases, individuals with Long COVID have reported improvements in their symptoms when treated with immunosuppressive therapies commonly used in autoimmune diseases. This suggests that modulating the immune response may be a potential approach to managing Long COVID symptoms.

**Figure 2:**
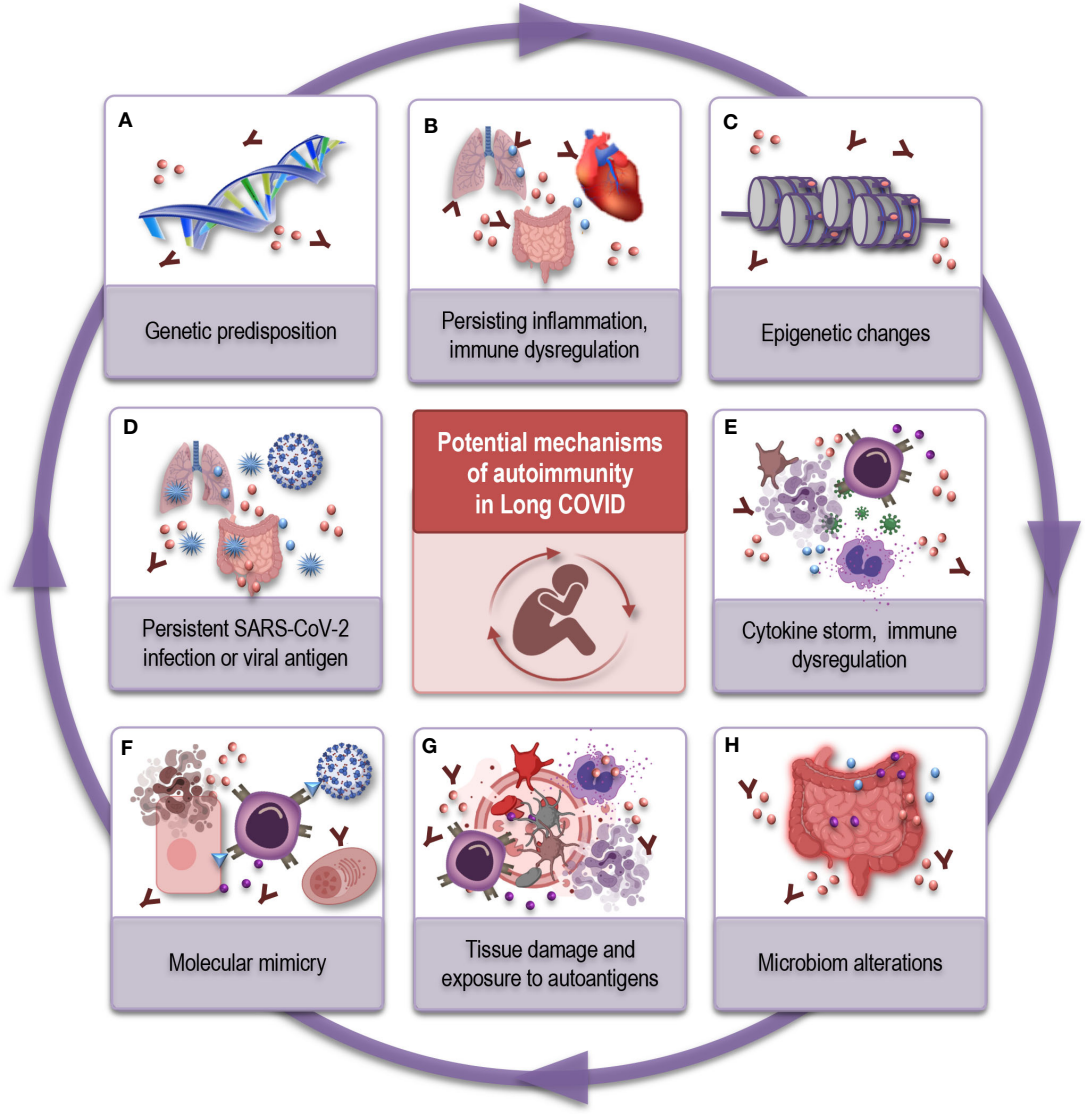
Possible mechanisms underlying autoimmunity in Long COVID. **(A)** Genetic predisposition: Genetic factors can influence an individual’s susceptibility to autoimmune diseases and their response to viral infections. Certain genetic variants may increase the risk of autoimmune-like reactions in Long COVID. **(B)** Persisting inflammation and immune dysregulation: Prolonged immune activation can cause chronic inflammation and autoimmune-like symptoms. **(C)** Epigenetic changes: SARS-CoV-2 infection may induce epigenetic modifications can contribute to autoimmune-like responses. **(D)** Viral persistence or reservoirs: After recovering from COVID-19, the virus or viral antigen might linger in the body in a dormant state, keeping the immune system activated, potentially leading to immune dysregulation and autoantibody production. **(E)** Cytokine storm and immune activation: Severe COVID-19 cases often involve an excessive release of inflammatory cytokines. Persistent immune activation and high cytokine levels can disrupt immune function, potentially leading to autoimmune reactions. **(F)** Molecular mimicry: Similarities between viral proteins and human proteins can result in autoimmune-like responses. **(G)** Tissue damage and exposure to autoantigens: COVID-19 can cause significant tissue damage, exposing autoantigens to the immune system. **(H)** Microbiome alterations: SARS-CoV-2 infection could disrupt the balance of the gut microbiome, affecting immune regulation. This imbalance may contribute to autoimmune-like responses. SARS-CoV-2, severe acute respiratory syndrome coronavirus type 2; COVID, coronavirus disease.

As mentioned above, the Long COVID is a multifaceted condition and the mechanisms underlying autoimmunity in Long COVID are complex and not fully understood (72, 73, 75). Several possible mechanisms have been considered and are summarized in **Figure 2** that may contribute to the development of autoimmune-like features in Long COVID patients.

One hypothesis is that SARS-CoV-2 may persist in the body in a dormant state after the acute infection. This prolonged viral antigen presence could continually stimulate the immune system, leading to inflammatory conditions, immune dysregulation, and the development of autoantibodies (**Figure 2D**). In some individuals, this immune response may not return to its normal state even after the virus is cleared, leading to chronic inflammation and autoimmunity (**Figure 2B**) (44, 71, 75, 76). The concept of inflammaging, characterized by chronic low-grade inflammation, resonates with the persistent inflammation encountered in Long COVID patients. In both scenarios, the inflammatory milieu could play a dual role: contributing to the manifestation of autoimmune phenomena in the aged, and serving as a potential driver of the immune perturbations and lingering symptoms in Long COVID patients. The overlap in inflammatory signatures between immunosenescence and Long COVID permit consideration of shared molecular mechanisms and need further research.

Molecular mimicry is another phenomenon - where viral proteins might share similarities with human proteins (**Figure 2F**). SARS-CoV-2 was shown to share epitopes with human proteins and this cross-reactivity could therefore trigger such autoimmune responses (77–79). Additionally, SARS-CoV-2 infection could potentially disrupt the balance of the microbiome (**Figure 2H**), which is crucial in immune regulation (80, 81). Dysregulated immune cells, such as T- and B cells, may contribute to the production of autoantibodies.

The cytokine storm, characterized by an excessive release of pro-inflammatory cytokines, is a main feature of severe COVID-19. Persistent immune activation and high levels of inflammatory cytokines can lead to immune system dysfunction and potentially autoimmune reactions (**Figure 2, E**) (44, 82). Additionally, during the acute phase of COVID-19, the virus can cause significant tissue damage exposing self-antigens to the immune system (44, 83) and leading to the induction of autoimmune responses (**Figure 2G**). And finally, SARS-CoV-2 infection may induce epigenetic changes in immune cells (84, 85), altering their function and potentially promoting autoimmunity (**Figure 2C**). Age-related epigenetic changes may also synergize with the post-viral outcome, potentially catalyzing immune dysregulation and propagating the symptomatology of Long COVID (85, 86).

Thus, individuals with different genetical (**Figure 2A**), immunological, and environmental background may experience conditions that are generated by interplay of distinct mechanisms or a combination of them. Therefore, the mechanisms mentioned above are not mutually exclusive, and multiple factors may interact to produce the autoimmune-like symptoms observed in some Long COVID patients.

Autoimmunity and Long COVID may intricately be intertwined, as demonstrated by the potential mechanisms discussed above. However, it is also worth mentioning that patients suffering from Long COVID sometimes present with symptoms resembling those seen in autoimmune diseases, despite the absence of identifiable autoimmunity markers using conventional laboratory methods. This phenomenon is not uncommon, and intriguingly, many of these patients do not display markedly elevated levels of inflammation. While SARS-CoV-2 can indeed trigger full-blown autoimmunity through the mechanisms elucidated earlier, there may exist other, yet-to-be-understood pathways that lead to low-grade inflammation. These pathways could serve both as pathogenetic mechanisms and manifestations of autoimmune or autoinflammatory disorders in the context of Long COVID. This intriguing observation underscores even more the complexity of Long COVID and the intricate relationships between viral infections, immune dysregulation, and the potential for autoimmune-like symptomatology.

Thus, this emerging area of research holds promise for uncovering novel mechanisms underlying immune dysregulation in Long COVID, which could shed light on the enigmatic symptoms experienced by patients. It also underscores the need for more refined diagnostic tools capable of detecting subtle immune alterations that may not be captured by conventional laboratory tests. This avenue of exploration promises to expand our understanding of Long COVID and its intersection with autoimmunity, offering hope for improved management and therapeutic interventions for affected individuals.

## 7 Conclusions

While the autoimmune component in Long COVID is still an area of active research, it is becoming increasingly clear that dysregulated immune responses may play a significant role in the development and persistence of Long COVID symptoms. Recognizing this autoimmune component opens up new avenues for potential treatments and therapies, as drugs that modulate the immune system and manage autoimmune conditions may prove beneficial for some Long COVID patients.

In essence, this mini-review serves as a modest contribution and remains just a stepping stone in the grand journey of understanding the complex interactions in immunosenescence, inflammation, and autoimmunity. Our hope is that this concise overview stimulates continued exploration, paving the way for innovative interventions, and ultimately advancing the understanding and management of autoimmune disorders within the context of aging.

## Author contributions

LM: Conceptualization, Supervision, Visualization, Writing – review & editing. SD: Methodology, Visualization, Writing – original draft.
